# Access to assistive technology in Africa: findings from evidence-informed deliberative dialogues on trade and fiscal policies

**DOI:** 10.3389/fresc.2026.1866962

**Published:** 2026-08-05

**Authors:** Emiliana Maria Grando Gaiotto, Mariana Gabriel, Magali Favaretto Prieto Fernandes, Odara Gonzaga de Andrade, Natália Hedlund Jardim, Vinicius Delgado Ramos, Nathan Mendes Souza, Bruno Guedes Alcoforado Aguiar

**Affiliations:** 1 Centro Universitário Nossa Senhora do Patrocínio, Itu, Brazil; 2 Hospital das Clinicas HCFMUSP, Faculdade de Medicina, Universidade de Sao Paulo, Sao Paulo, SP, Brazil; 3 Faculdade Galileu, Botucatu, Brazil; 4 Universidade Federal de Minas Gerais, Belo Horizonte, Brazil; 5 Universidade Federal do Piauí, Teresina, Brazil

**Keywords:** assistive technology, deliberative dialogue, evidence-informed policymaking, public policy, tax exemption

## Abstract

Access to assistive technology remains unequal in many countries, particularly in low- and middle-income settings, where high costs, dependence on imports, and fiscal and trade barriers can limit availability and affordability. This article aimed to present and analyze deliberative dialogues conducted with participants from Mozambique, Chad, Egypt, and Kenya, informed by an evidence synthesis on reducing tariffs and taxes on assistive technology. Three multilingual deliberative dialogues were conducted between August and October 2025, involving 35 participants from different stakeholder groups. The dialogues were used as a knowledge translation strategy to contextualize scientific evidence through structured discussions among different stakeholders. Representatives from the Republic of the Congo were invited but did not participate. Transcripts were subjected to thematic content analysis based on Bardin's approach and organized according to the domains of the SUPPORT tools. The findings revealed common challenges, including high costs, dependence on imports, weaknesses in the implementation of tax exemptions, limitations in local production, territorial inequalities, shortages of specialized professionals, and difficulties in coordination across sectors and institutions. Country-specific issues related to rural access, governance, territorial inequalities, device acceptability, and policy implementation were also identified. The options discussed included fiscal and tariff reforms, strengthening local production, international cooperation, and awareness campaigns. The deliberative dialogues enabled the contextualization of evidence across different national settings and demonstrated that expanding equitable access to assistive technology requires combined strategies adapted to the specific characteristics of each context.

## Introduction

Assistive Technology (AT) is based on the use of assistive products, which the ISO 9999:2022 standard defines as any product—including devices, instruments, equipment, and software—intended to optimize a person's functioning and reduce disability. According to the standard, these resources, whether specially produced or generally available, act as essential environmental factors to mitigate activity limitations and participation restrictions (1). The World Health Organization (WHO) and the United Nations Children's Fund (UNICEF) include AT among actions aimed at rehabilitation, universal health coverage, and expanding social participation (2).

Access to AT is also part of the commitments made by countries under United Nations Convention on the Rights of Persons with Disabilities which provides for the adoption of measures to expand the availability of and access to these products and services (3). This agenda aligns with the Sustainable Development Goals, especially those related to health, education, employment, reducing inequalities, and strengthening public institutions (4).

According to the Global Report on Assistive Technology, more than 2.5 billion people are estimated to need at least one assistive product, and this number is projected to exceed 3.5 billion by 2050. At the same time, approximately one billion people still cannot access the products they need. The largest gaps are concentrated in low- and middle-income countries, where limitations related to financing, product supply, availability of qualified professionals, and the organization of services reduce access to AT (2).

In Africa, these difficulties are compounded by dependence on imported products, limited local production capacity, and poorly structured supply chains. As a result, many products remain unavailable or reach users at costs incompatible with the economic reality of the population. Furthermore, differences in tax regimes, customs procedures, and international classification of goods hinder both trade monitoring and the implementation of policies aimed at expanding access (2, 5).

These aspects correspond to environmental factors described in the International Classification of Functioning, Disability and Health (ICF), which recognizes the influence of public policies, health systems, the physical environment, products, and economic conditions on people's functioning and participation (6). Among these factors, this study focuses on trade and fiscal policies, including import tariffs, taxation, and the classification of goods by Harmonized Commodity Description and Coding System (HS), used internationally for customs and statistical purposes (7).

Although access to AT has been discussed from different perspectives, studies that specifically address trade and fiscal policies are still limited. To organize the available knowledge, Gaiotto et al. (8) They conducted a literature review on the topic, identifying existing scientific production and highlighting gaps related to the use of tax, trade, and regulatory policies to expand access to AT. This survey supported the development of a synthesis of evidence subsequently published by Gabriel et al. (9),which gathered and critically analyzed the best available evidence on policy options, implementation strategies, equity aspects and barriers related to tariff and tax reduction, strengthening local production, international cooperation and improving regulatory mechanisms.

Making this evidence available represents only one step in the policy-making process. Its use depends on mechanisms capable of integrating research results with the institutional, social, and political realities of each context. In this sense, Deliberative Dialogues constitute a knowledge translation approach used to promote discussions among researchers, managers, professionals, representatives of civil society, and other actors involved in decision-making (10–13).

Despite the increasing use of Deliberative Dialogues in different areas of health, studies describing how evidence syntheses on trade and fiscal policies related to AT are contextualized and used in deliberative processes, particularly in African countries, remain scarce.

This study corresponds to the subsequent stage of this research program and aimed to analyze the results of the Deliberative Dialogues held with representatives from Mozambique, Chad, Egypt, and Kenya, developed from the synthesis of evidence published by Gabriel et al. (9). The study sought to understand how evidence was discussed, contextualized, and incorporated into reflections on public policy options aimed at expanding access, equity, and sustainability of AT systems in different national contexts.

## Methodology

### Study design

This study corresponds to the second stage of a knowledge translation project aimed at strengthening evidence-informed public policies to expand access to AT in African countries. The project was structured in two complementary stages. The first consisted of developing a synthesis of evidence on trade and fiscal policies related to AT, built from a previously published literature inventory (8) and subsequently synthesized by Gabriel et al. (9). This synthesis identified policy options, barriers, facilitators, and aspects related to implementation and equity, constituting the technical material used in the Deliberative Dialogues.

The second stage involved conducting Deliberative Dialogues (DD), used as a knowledge translation strategy to discuss and contextualize the evidence produced in the previous stage. Although both phases are part of the same project, this article presents exclusively the methods and results related to the Deliberative Dialogues.

The study was conducted using the SUPPORT (SUPporting Policy Relevant Reviews and Trials) tools, developed to support the systematic use of scientific evidence in public policy formulation, simultaneously considering scientific, institutional, social, economic, and political factors (10, 14). The approach also followed the principles of the World Health Organization's Evidence-Informed Policy Network (EVIPNet), which promotes the integration of scientific evidence and contextual knowledge to support health decision-making (13).

### Study context

The Deliberative Dialogues were developed within the scope of a project coordinated by Instituto de Medicina Física e Reabilitação (IMREA), with funding fromATscale—Global Partnership for AT, with the aim of supporting the formulation of public policies aimed at expanding access to AT in African countries through the use of scientific evidence and knowledge translation strategies.

The participating countries were defined during the project planning phase, considering the criteria established by ATscale to encompass different African contexts regarding the level of development of the AT sector and the maturity of policies related to international trade. Furthermore, the aim was to include different regions of the continent and linguistic contexts (Portuguese, French, and English), favoring the discussion of evidence in distinct scenarios. Thus, the following countries were selected…Mozambique, Chad, Egypt, Kenya and the Republic of Congo, without claiming to represent all African countries.

Although the Republic of Congo was included in the planning stage and invited to participate in the Deliberative Dialogue held in French, no representatives were present at the meeting. Therefore, the analyses presented in this study refer to the dialogues held with representatives of…Mozambique, Chad, Egypt and Kenya.

### Participants

The participants were intentionally selected to bring together different perspectives related to access to AT. Invited participants included public administrators, researchers, health and rehabilitation professionals, representatives of civil society organizations, organizations of people with disabilities, international institutions, and other actors involved in the formulation, implementation, or monitoring of policies related to AT.

Invitations were extended based on technical expertise, institutional performance, and participation in AT initiatives in their respective countries. Only participants who accepted the invitation, signed the Informed Consent Form, and actively participated in the Deliberative Dialogues were included in the analysis.

### Data collection

The Deliberative Dialogues were held between August and October 2025, in a virtual environment, using the Zoom platform. Three dialogues were conducted: Mozambique (August 22, 2025), in Portuguese; Chad (September 12, 2025), in French; and Egypt and Kenya (October 3, 2025), in English. The meetings were scheduled to take place from 8:00 AM to 12:00 PM (Brasilia time).

Thirty-five representatives participated in the dialogues, including government managers, health and rehabilitation professionals, researchers, representatives of civil society organizations, organizations of people with disabilities, AT users, and representatives of international organizations, according to the composition of each country. The Mozambique dialogue had 11 participants; the Chad dialogue had 8 participants; and the joint dialogue between Egypt and Kenya brought together 16 participants, with 14 representatives from Kenya and 2 from Egypt. Congo was also invited to participate in the dialogue, which was conducted in French; however, no representatives were present, which is why this country was not included in the analyses.

Before the dialogues took place, all participants received the summary of evidence published by Gabriel et al. (9). The document, containing a description of the problem, five policy options, implementation considerations, and equity-related aspects, served as the basis for discussions and was used by all participants during the deliberative process.

The dialogues were led by a moderator with experience in knowledge translation, supported by a team responsible for recording the activities. The dialogues followed a structured script based on the SUPPORT tools, encompassing four discussion domains: problem, policy options, equity, and implementation. These domains guided both the conduct of the dialogues and the organization and analysis of the data.

With the participants' permission, all meetings were recorded. The recordings were subsequently transcribed using an artificial intelligence-based tool, and the transcripts were fully reviewed by the researchers by comparing them with the original audio recordings to ensure the accuracy of the information used in the analysis.

### Conducting deliberative dialogues

The Deliberative Dialogues were structured according to the recommendations of the SUPPORT tools and organized into four discussion domains: problem, policy options, equity, and implementation.

The discussions were guided by five policy options previously identified in the evidence synthesis: (1) tariff reduction through simplified procedures for exemption and preferential tariffs applied to AT products; (2) improvement of national tax structures; (3) strengthening local production through incentives, financing and regional integration; (4) expansion of international partnerships and cooperation; and (5) development of awareness campaigns on AT.

This structure was used to organize the debates and also guided the subsequent data analysis stage.

### Data analysis

The data from the transcripts of the Deliberative Dialogues were analyzed using content analysis, according to the methodological framework proposed by Bardin (15). Initially, a preliminary reading of all the material was conducted to familiarize oneself with the analytical corpus. Following this, the transcripts were organized according to the four domains that structured the Deliberative Dialogues, as per the SUPPORT tools: problem, policy options, considerations regarding implementation and considerations on equity.

Subsequently, the material was coded and categorized to identify the main themes discussed by participants across the SUPPORT domains. Based on this process, an analytical synthesis was developed and presented in the *Analytical synthesis* column. This synthesis summarizes the main issues discussed, areas of consensus and divergence, and the principal recommendations emerging from the deliberative dialogues. For each analytical synthesis, illustrative excerpts from participants' statements were selected and presented in the *Illustrative quotations* column to exemplify and substantiate the findings.

The analysis was conducted by one researcher with experience in evidence synthesis and qualitative analysis, who was responsible for coding and interpreting the data. The analysis was subsequently reviewed by a second researcher. Any differences in interpretation were discussed between the two researchers until consensus was reached, aiming to enhance the consistency and reliability of the analytical process.

### Ethical aspects

All participants signed the Informed Consent Form before the start of the Deliberative Dialogues. Contributions were treated confidentially and analyzed anonymously. The study did not involve the collection of clinical data, intervention with participants, or sensitive personal information.

## Results

The results are presented in analytical matrices organized according to the SUPPORT domains. For each domain, the *Analytical synthesis* column presents the synthesis developed from the content analysis of the discussions, including the main themes discussed, areas of consensus and divergence, and the principal recommendations emerging from the deliberative dialogues. The *Illustrative quotations* column presents representative excerpts from participants' statements that support and exemplify the analytical synthesis. Each matrix corresponds to a Deliberative Dialogue conducted in a specific national context, allowing the presentation of country-specific findings while facilitating comparison across the different settings analyzed.

### Mozambique

Table 1 presents a synthesis of the deliberations from the Deliberative Dialogue held in Mozambique. The results are organized according to the four SUPPORT domains: problem, policy options, implementation considerations, and equity considerations. For each domain, the matrix presents an analytical synthesis of the discussions, accompanied by illustrative quotations from participants that exemplify the main findings identified through the content analysis.

**Table 1:** Summary of the deliberations of the deliberative dialogue held in Mozambique, organized according to the domains of the SUPPORT tools.

SUPPORT domain	Analytical synthesis	Illustrative quotations (anonymous statements from stakeholders)
Problems	Fragile local production	“*The centers that should be producing, unfortunately, are not producing.”* “*…as long as we continue to depend heavily on this material…it will always be complicated to produce locally.*” “*…the maintenance of the equipment itself is also related to the acquisition of consumables…*” “*Although we have personnel and several centers, at least one in each province, as long as we don't have the materials, it will always be difficult to produce locally.”*
	Cost to the user	“*Wheelchair users in Mozambique have very limited employment opportunities, and the cost of a wheelchair is very high. In terms of price, it is around four minimum wages, which is unaffordable for people with limited employment opportunities.”* “*Most assistive products are only available in the private sector. They are practically unavailable in the public sector, where they would likely be less expensive. When they are available in the public sector, they are usually donations.”* “*…not to mention the low quality of the wheelchairs sold here. Or when they are sold, they are unsuitable for everyday use.”* “*…the purchase of the product ends up being expensive for the population…”*
	Health priorities	“*…we are battling malaria, tuberculosis…investment in assistive technology is not considered a priority.”* “*The government's priority right now is really to look at mortality rates, not quality of life.”*
	Limited training and specialized workforce	“*We don't have a rigid or standardized training program for professionals in the field of assistive technology.”* “*There is a very large gap in human resources in this specific area.”*
	Geographic access	“*The population is spread across a very vast territory.”* “*The access routes are not very favorable for the use of assistive technologies.”* “*…not all buildings are prepared…this is still a challenge…”* “*…how is this patient going to get to the health unit…”*
	Limited awareness of assistive technology	“*…assistive technologies are still considered something new in our context”.* “*…there is a lack of knowledge…about the difference in a person's life between having assistive technology and not having that technology.”* “*…there is a lack of knowledge"…and how this assistive technology can help other areas…”*
	Limited connectivity	“*…there are places where the internet doesn't reach with any quality…that population doesn't have access to technology…”*
	Limited psychosocial support	“*…the users have a medical diagnosis, but they are not followed up from a psychosocial perspective…this can be problematic in the rehabilitation of this user.”*
Option 1	Reduce/eliminate tariffs and non-tariff barriers; ’Single Window’; data/AI-driven customs risk management; regulatory harmonization and memoranda of understanding (MoUs) with customs authorities	“*…I would opt for effectively reducing tariffs through simplified procedures…the cost doesn't just come from the acquisition, but from all the bureaucracy involved in reaching the user. The price skyrockets…”* “*For me, option one would be better…reducing the tariffs.”* “*…reducing the fees would not benefit patient access; it would benefit the institutions that sell these assistive technologies…this would not guarantee access for the people who really need it.”* “*…with import tariffs so high, the bureaucracy makes the final price exorbitant. Reducing tariffs could facilitate access to quality products.”* “*…as an association we tried to import some wheelchairs through a donation from a foreign entity, but we were unable to because we couldn't obtain the exemption…”*
Option 2	Simplify value-added tax (VAT) exemptions/refunds, positive lists, portals, and automated prescription align taxation with essential services; combine subsidies/vouchers with social protection.	“*The exemptions provided for don't work at the operational level; the processes are opaque and time-consuming.”* “*…public policies should provide partnerships so that people who lack purchasing power can access services at a reduced price or for free.”*
Option 3	Co-creation with users; maintenance/reuse workshops; local production/assembly with incentives; capacity building (inclusive design); regional coordination for supplies and scale.	“*…local production is one of the alternatives that we are seeing as viable for the long-term maintenance…promotion of local production of products…”* “*…requesting supplies from one hospital to another…”* “*…we have machines, we have the possibility of recycling some materials for local manufacturing.”* “*…with mass production capacity, there would be a greater possibility for more patients to have access.”* “*…local production is important, but it will take time to be developed.”* “*…if there were incentives for funders, it would be much easier to produce assistive technology in Mozambique.”* “*…from my own experience, I would opt more for local production, as it is more accepted and more widely publicized at the local level…”*
Option 4	International partnerships	*“We already had a partnership with Handicap International and the Ministry of Gender… patients were identified at community level, referred to the hospital, evaluated and received the assistive technology they needed.” “…a parceria para nós seria importante…”* “*…to promote international cooperation partnerships…to strengthen what governmental activities often fail to achieve…”*
Option 5	Public awareness and community engagement	“*Reduce stigma, increase active outreach and identification of people with disabilities who do not access assistive technology services/assisted therapy”* “*Campaigns reduce stigma.”* “*…to strengthen awareness in the communities…”* “*…one of the programmes with the greatest impact and uptake was community-based rehabilitation.”* “*…the concept of disability still carries considerable stigma and discrimination.”* “*…we still find Mozambican families hiding people with disabilities because of lack of information.”* “*…these campaigns can greatly facilitate access to assistive technology.”* “*…we still have many people with disabilities hidden due to lack of diagnosis and awareness…”*
Considerations of Implementation	Missing flows/protocols	“*…lack of identification or a flowchart criterion for the allocation and use of assistive technologies.”* “*…there is no follow-up on the adaptation and rehabilitation process.”* “*…often this adaptation has not been done rigorously…”* “*…it's not just about someone deciding to deliver a technology. It's necessary to consider whether it's acceptable, feasible, and whether the person will actually use it…”* “*…one of the barriers would be the lack of flowcharts for the care or distribution of assistive technologies.”* “*…sometimes they are there, but they are not directed to where they should acquire or be observed for assistive technologies.”*
	Governance	“*…it's a problem at all levels: central, provincial…”* “*…there are provinces that have supplies but no patients…others have many patients but no supplies…”* “*In Mozambique, the approval of Law 10/2024 represents progress, although its practical implementation presents challenges.”* “*…we have laws that allow people with disabilities to import certain materials, but in practice this hasn't been happening.”* “*…there are norms, rules…but they are not being followed.”* “*Organizations of people with disabilities (DPOs/FAMOD and associations) know best where the users are located and should participate more in the implementation of policies.”*
	Digital health and telemedicine	“*…telemedicine can be used as one of the facilitators for healthcare workers.” “…integration of services using telemedicine to expand knowledge.”* “*…resistance to change…”*
	Maintenance cost	“*…the cost of maintenance…”*
	Dependence on external suppliers	“*…dependence on external suppliers…”*
	Limited referral knowledge	“*Healthcare workers need to be aware. Because there are some who, when assessing people, are unaware of who they should refer them to for the proper evaluation and prescription of assistive technology.”*
Equity Considerations	Underestimation of prevalence rates	“*…many times the association of people with disabilities strongly disputes the percentage of disabled people we have in the country…this figure is a bit underestimated.”* “*Psychosocial conditions have worsened post-pandemic; 2017 census underestimates this.”* “*…they suffer stigma, they suffer discrimination…”* “*…people often associate disability with some kind of curse…and end up hiding their family members with disabilities.”*
	Equitable distribution	“*…equitable distribution, also considering the different regions of our country.”* “*…there are provinces, such as Cabo Delgado, that do not have a prosthetic laboratory.”*
	Unequal access to opportunities	“*…we found several 14 and 15-year-olds who had never been to school due to a lack of assistive technology…”*
Dialogue guidelines	To strengthen advocacy, awareness, and the dissemination of knowledge about assistive technologies among decision-makers and society.	“*…not everyone knows about assistive technology. So, if we don't talk about it, fight for it, and advocate for it, nobody will know about it.”*
	Strengthen intersectoral collaboration	“*…this dialogue reinforces the need for synergies…”*
	Local production	“*…I will reiterate that the sustainability of local production is very important and urgent in Mozambique.”*
	Regulation of laws	“*…the policies exist in Mozambique, but there is a lack of regulation of these policies…”*
	Campaigns	“*…I will conclude by emphasizing the component of awareness campaigns…”*

Source: Author's own elaboration.

### Chad

A Table 2. This presents a summary of the deliberations from the Deliberative Dialogue held with representatives from Chad. Initially, the participation of representatives from Chad and Congo was planned for the dialogue, which was conducted in French; however,It was not possible to secure the participation of representatives from Congo.Therefore, the results presented refer exclusively to the contributions of the participants from Chad. The matrix is organized according to the domains of the SUPPORT tools.

**Table 2:** Analytical matrix of the deliberative dialogue held with representatives from Chad, organized according to the domains of the SUPPORT tools.

SUPPORT domain	Analytical synthesis	Illustrative quotations (anonymous statements from stakeholders)
Problems	Limited rehabilitation services	“*The country's first Physical and Rehabilitation Medicine unit has just been established.”* “*Unfortunately, there is no assistance, and these people are left behind.”* “*It is a serious problem for our country.”* “*Even customs services are unfamiliar with some of the products we use, so I first have to explain them.”* “*There is also a lack of awareness; information does not reach beneficiaries who are persons with disabilities.”*
	Need for assistive technology	“*It remains a real problem for people who need assistive technology.”* “*First, gaining access to mobility aids, wheelchairs and crutches is already a problem.”* “*Indeed, there is an accessibility problem, not only financial, but accessibility in every sense.”* “*When I offer them more advanced technology, it comes as a surprise to them.”* “*The needs are there […] On the ground, there is a serious need for assistive technology.”* “*There is a real need, and this need […] is a national problem.”* “*Most of the products that could really make an impact cannot be found [in the finance law].”* “*Patients are already travelling thousands of kilometres to reach the service.”* “*How can these wheelchairs be transported to other regions, especially when some regions are unable to manufacture them?”*
	High cost	“*For deaf people today, obtaining a hearing aid requires more than one million [CFA francs]. How many people can actually afford that?”* “*A wheelchair is not affordable for everyone.”* “*You can buy something for two dollars […] but by the time you receive it locally, it costs five dollars. Imagine how difficult that makes it to provide these assistive products.”* “*Assistive products are brought in […] but the cost is excessively high.”* “*People are extremely poor.”* “*We need to consider how to make assistive technology accessible to the most vulnerable people, including persons with disabilities.”*
	Limited technical workforce	“*I am still the only specialist.”* “*There is a need to increase the qualified technical workforce. […] this is a major concern: he is alone.”* “*Many things are being done, but each actor works separately […] making it difficult to consolidate and build on these efforts.”* “*There is a critical shortage of professionals in the field […] this is a major barrier to access to assistive technology.”* “*I am trained as a prosthetist and orthotist […] I walk with crutches because I cannot obtain the type of prosthesis I need in Chad.”*
	Lack of assistive products	“*We need the necessary equipment to properly support these patients.”* “*The wheelchairs manufactured here in Chad are not appropriate.”* “*All of this already raises problems of quality.”* “*Its use can have negative effects on the human body.”* “*There is also the issue of adaptability to the terrain and to the individual users themselves.”* “*What happens here is that local production does not comply with these basic technical principles.”* “*I had to use splints that were not custom-made […] I had to heat the plastic and somehow reshape them myself.”* “*I would really like these products to be available, because they are needed.*”
	Conflict-related disability	“*It is a real public health problem, since the country has gone through several years of war, leaving many people with physical impairments.”*
	Late diagnosis and disability	“*Today, chronic diseases are diagnosed quite late and managed late, resulting in sequelae and disabilities.”*
	Trauma-related disability	*“The leading cause of emergency consultations is road traffic trauma, as well as injuries involving bladed weapons.”*
Option 1		“*If they handle procurement, supply, dispensing, and distribution should become easier, and access should become more affordable.”*
Option 2	Product codification	“*These products are not identified and codified in the finance laws.”* “*There is a need for an official document listing assistive products for persons with reduced mobility.”* “*We would like assistive products to be integrated into Chad's list of essential medicines and health products.”* “*Lack of awareness of tax exemption regulations.”*
Option 3	Local production	“*Local production needs to be improved through training and technical support.”* “*Ergonomic principles must be fully respected, as this is what will positively affect the user's life.”* “*The qualified technical workforce needs to be increased.”* “*People should be given the opportunity to produce products locally in order to reduce costs.”* “*Option three will first require political commitment and financing […] particularly in Chad. This could be a somewhat difficult situation.”*
Option 4	Partnerships/cooperation	“*Under option four, we can use options one and two as areas of action through which partnerships can develop effective work.”* “*The World Health Organization, as a leader in health, has taken up the issue and recognized it as a public health concern.”* “*We have other partners with years of experience who have lessons learned to share.”* “*Partners with experience in these areas can provide technical support to national actors to develop these technical tools.”* “*To establish cooperation partnerships […] with countries that have made advances in assistive technology.”* “*First, we need to start with partnership—option four.”* “*In terms of priority, option four comes first, followed by options one and two with the support of option four. Then option three follows, ending with option five.”*
Option 5	Awareness/advocacy	“*The laws granting these benefits need to be widely disseminated and publicized.”* “*I would give much greater priority to option five, because if people do not understand what assistive technology is, it will be difficult to implement it and communicate the message.”* “*For me, option five remains the driving option for addressing the other options.”* “*What was missing from the options presented here was the priority of having local data—local evidence.”* “*The priority is first to ensure that everyone understands the issue and that everyone has the same level of information.”* “*The first thing to do is to challenge stereotypes and negative perceptions about disability through awareness-raising, advocacy, and similar actions.”* “*Yet needs are accumulating […] we must use option five for advocacy in order to advance options one and two.”* “*What was missing from the options presented here was the priority of having local data—local evidence.”* “*The priority is first to ensure that everyone understands the issue and that everyone has the same level of information.”* “*With all stakeholders, because this is not the responsibility of a single sector, but of several sectors.”* “*Awareness-raising should not focus only on direct beneficiaries; political authorities should also be targeted.* *The first thing to do is to challenge stereotypes and negative perceptions about disability through awareness-raising, advocacy, and similar actions.”* “*It is therefore necessary to immediately begin with awareness-raising and advocacy […] and make authorities understand the need for assistive technology in Chad to support the autonomy of persons with disabilities.”*
Implementation considerations	Governance	*The Ministry of Health recognizes TA as a priority and informs that it is included in the National Health Policy and the National Health Development Plan (2022–2030).* “*Now, the authorities will need to support us.”* *There is a lack of knowledge about assistive technology among health professionals, users, and the government* “*The fact that health professionals are now with us and approaching the issue positively is also a facilitating factor.”* “*Organizations of persons with disabilities should also take ownership of the issue and move it forward.”* “*Through the Ministry, we have a focal point […] this shows that assistive technology is already a concern for the Chadian government.”* “*The country's laws and regulations are not widely known, even among decision-makers.”* “*The lack of information at both government and population levels is a major concern.”* “*When assistive devices are prescribed, part of the cost should be covered by the country.”* “*We have a representative within the Ministry of Health, a focal point who is already working on this issue.”* “*If you look at strategic documents and health development plans, disability may be addressed in only two or three paragraphs.”* “*When public institutions do not have strategic plans addressing assistive technology and disability, this becomes a difficult barrier to overcome.”* “*Although political will exists, the lack of strategies at the operational and implementation levels poses a real problem.”* “*We would not want private-sector actors to capture the tax benefits and profit from them.”* “*We advocated for assistive technology to be included in universal health coverage and to be subsidized or supported, but it was not included.”* “*There are also focal points and dedicated departments […] including within the social affairs sector.”*
	Lack of needs data	“*We have requested support from partners to conduct an assessment of all needs, in order to obtain data that can support advocacy.”* “*To have data that can enable us to advocate and mobilize resources, including domestic resources.”* “*For us, this remains a primary concern […] this should be done to give us the means to demonstrate the situation.”* “*To use [the evidence] as a means for advocacy or awareness-raising.”*
	Awareness	“*Once everyone agrees that this is important and makes it a priority […] each actor can take responsibility for the option within their area.”* “*The country faces many of these situations, but insufficient information is provided to the different groups and stakeholders.”* “*There is misunderstanding within financial services regarding customs duty reductions.”* “*The engagement of organizations of persons with disabilities is a positive achievement that already exists.”* “*We professionals are already engaged with these issues. This is a good starting point for us.”* “*It is necessary to communicate with healthcare providers and prescribers to inform them that these products are available.”*
	Limited institutional coordination	“*There is insufficient interaction between public services, the focal point, and associations.”* “*Out of 23 provinces, only two have rehabilitation device services. The other provinces have none.”* “*We should come together in synergy and determine what can be done through each professional profile.”*
	Tax exemption	“*Products intended for persons with disabilities are, for example, exempt from taxes.”* “*How can we ensure that this information reaches those who can help with imports?”*
	Insufficient advocacy	“*Insufficient advocacy can clearly be a barrier to this process.”*
	Financial protection	“*If state social services can provide coverage, beneficiaries will be better able to obtain assistive devices.”*
	Geographic disparities in access	“*The capital N'Djamena may be better served than the provinces […] may benefit more than other regions.”*
Equity considerations	Geography	“*Generally, N'Djamena has access to certain devices. When you go further into the provinces, the situation changes.”*
	Socioeconomic	“*Poverty prevents beneficiaries from directly obtaining these devices.”*
	Information/stigma	“*The major problem in Chad is the lack of awareness about disability and, especially, assistive technology. People are not aware of it.”* “*They say that disability is a curse from God, and people do not pay attention to those affected at all.”*
	HR	“*Severe shortage of professionals; concentration in the capital.”* “*There is one physical and rehabilitation medicine physician for 18 million people, barely 20 prosthetists and orthotists, barely 15 physiotherapists, and no speech therapist.”* “*We already have a foundation of fewer than 100 professionals. This is already an asset.”*
Dialogue guidelines	Develop a workforce expansion plan	“We can develop a plan to expand the workforce. It is possible; it is not impossible.”
	Next steps	“With everyone's collaboration, this issue can truly gain momentum […] and this momentum should continue.” “Thank you for enabling all stakeholders, especially Chadian stakeholders, to come together. I believe this is a first step, but a major one.”

Source: Author's own elaboration.

### Kenya and Egypt

The Deliberative Dialogue with representatives from Kenya and Egypt was held in a single session, conducted in English. However, during the analysis, the contributions were organized separately by country, considering the differences in institutional, regulatory, and operational contexts reported by the participants.

Table 3 presents a synthesis of the deliberations of the Egyptian representatives who participated in the Deliberative Dialogue conducted jointly with participants from Kenya. Although representatives from both countries took part in the same dialogue, which was conducted in English, the results are presented in separate tables to preserve the contributions and contextual specificities of each country. The results are organized according to the four SUPPORT domains: problem, policy options, implementation considerations, and equity considerations. For each domain, the matrix presents an analytical synthesis of the discussions, accompanied by illustrative quotations from participants that exemplify the main findings identified through the content analysis.

**Table 3:** Analytical matrix of the contributions of the Egyptian representatives in the deliberative dialogue held jointly with representatives from Kenya, organized according to the domains of the SUPPORT tools.

SUPPORT domain	Analytical synthesis	Illustrative quotations (anonymous statements from stakeholders)
Problems	High cost of assistive products	*“We have a very big problem dealing with assistive devices, especially prostheses. It's very, very expensive.”*
	Reliance on assistance and charity	*“People with disabilities who need this must go […] to get it free, or get charities. It's not easy to afford it. It's very expensive.”*
	Limited availability of advanced technologies	*“People who have money to afford [it], they go out of Egypt to bring […] assistive devices with new technologies that are not here in Egypt.”*
	High demand for assistive products	*“We have common diseases […] as diabetes, with patients with diabetic foot undergoing amputation, road traffic accidents, cancers […] that need amputation.”*
	Insurance coverage gaps	*“The problem encountered with that is population [who] are not working […] not even in the private sector.”* “*Most patients who come to a university hospital are not covered with insurance.”*
Option 1		*Not specifically discussed*
Option 2		*Not specifically discussed*
Option 3		*Not specifically discussed*
Option 4	Expanding national and international cooperation	*“Maybe after this dialogue, we can also refer nationally or internationally.”* “*We hope […] to make an association, international association.”* “*In UNICEF catalog, it's actually sold at $115. That price you can never find anywhere globally, and that is part of UNICEF and WHO effort to reduce those prices of those hearing aids.”* “*If the government of Kenya or the government of Egypt want to buy that product through UNICEF, they will get it at that price.”*
Option 5		*“If the health worker […] is under-trained or with low facilities, it will damage the whole things.”* “*If the health workers is […] well-trained, well-educated, well-talented, aware […] how to make each disabled people take his right fully.”*
Implementation considerations	Insurance coverage for specific groups	*“The insurance of Egyptian population is covering people from zero age to 18. This is the school insurance. And who attend any university […] also covered by insurance.”* “*The insurance […] accommodates the need of assistive devices, even prostheses, auditory aids, whatever.”*
	Governance	*“The government introduced [a] program […] disabled people who provide papers that [they are] not covered by insurance […] take the benefit from Takaful and Karama.”* “*The social associations also play a great role to collect money […] make deals to reduce the expenses of orthoses, auditory aids.”* “*If in the Ministry of Health, I would help, I could transfer to a charity, or mosque, or more higher.”* “*Just imagine a very strong health system […] The health provider or the health worker, or the manpower is not well-trained. So, what is the benefit?”* “*The government with the healthcare providers as a teamwork […] The interplay between the government and the healthcare providers.”*
	Religious and charitable support	*“We have […] holy organs like church and mosque that participate and collect that zakat.”* “*They go to the Takaful and Karama, charities, churches, mosques, to get money from collected money, and this is the pathway that they take.”*
	Guidance supports access to rights	*“We assist people […] to make them know their role, their right, the right way to take their benefit from each sector.”*
Equity considerations	Remote populations remain excluded	*“Still there are persons who are very far away […] from us or the government.”* “*The place of residence is very important. Here in Egypt, Cairo and Alexandria are two big cities with more facilities than other rural parts.”*
	Financial barriers shape inequities	*“Education, socioeconomic, they are linked to financial issue. So, financial issue is very important.”*
	Disability affects the whole family	*“If one person is disabled in the family, the whole family is suffering.”*
	Family support influences vulnerability	*“If he is lonely or has no family, or the only child, it will be very difficult.”*
	Gender may influence family burden	*“I want to give priority to male because […] in Egypt, or mostly most countries, the male is the one who […] take money.”*
Dialogue guidelines	*“Any solutions for me, uh, is perfect 100%. […] If they will be applicable to all of us, it will help to solve the need of disability persons.”*	

Source: Author's own elaboration.

Table 4 presents a synthesis of the deliberations of the Kenyan representatives who participated in the Deliberative Dialogue conducted jointly with representatives from Egypt. As in the previous table, the results are presented separately by country to preserve the contributions and contextual specificities of each national setting. The results are organized according to the four SUPPORT domains: problem, policy options, implementation considerations, and equity considerations. For each domain, the matrix presents an analytical synthesis of the discussions, accompanied by illustrative quotations from participants that exemplify the main findings identified through the content analysis.

**Table 4:** Analytical matrix of contributions from Kenyan representatives in the deliberative dialogue held with Egypt and Kenya, organized according to the domains of the SUPPORT tools.

SUPPORT domain	Analytical synthesis	Illustrative quotations (anonymous statements from stakeholders)
Problem	Limited insurance coverage	*“The few people who have insurance in Kenya […] the insurance does not cover the assistive devices.”*
	Assistive devices remain uncovered	*“Even when you are fully covered […] the insurance will cover so many other things, but not that assistive device.”* “*If you look at that fund, even after a lot of lobbying, they were only able to […] factor in very few devices.”*
	High out-of-pocket costs	*“But again, the amount is so huge that is expected out of you to pay.”* “*Getting that device is not easy, or even cheap, for me to purchase, or even hearing aids has also been very, very hard for me.”* “*There is a challenge in acquisition of assistive technology locally […] the issue of finance coming in within the accessibility devices.”* “*It's a big challenge as well, because most people are not able to afford these devices.”* “*Sometimes they go for the ones which are called amplifiers, which are not good for people with hard of hearing.”*
	Insufficient financial support	*“National Council of Persons with Disabilities do not cover everything. They will give you just a partial payment of that.”* “*If you look at the cost that has been pegged onto these assistive devices, it is not enough. For a wheelchair, Kenya shillings 15,000 will not be able to buy you a wheelchair.”*
	Difficulty obtaining assistive devices	*“Getting or purchasing some of these assistive devices, it's been very, very hard for me.”*
	Limited domestic availability	*“White cane […] is still very hard to get in Kenya. We import them.”*
	Dependence on imported devices	*“Getting organizations where you can buy them from, within the country or outside the country, it's very, very hard.”* “*I must just import it, because there's nowhere else I can get it apart from getting from maybe America or any other developed country.”* “*Even the tariffs put on them in order for us to purchase them is also very high for us.”*
Option 1	Unclear tax exemptions for AT products	*“What are some of the AT products that are actually recognized by the government? […] some of the vision products are not actually zero-rated.”* “*Maybe the government would need to clarify and maybe come up with a tentative list of what is zero rated and what is not zero rated.”* “*A tentative list of what is zero rated and what is not zero rated […] aligned with the new Act.”* “*We have laws or policies that promote exemption of these tariffs in Kenya currently.” But […] we still have barriers to access this, despite having these laws, which means the implementation is feeble, is weak.*
Option 2	Policies should harmonize access	*“You find that there is no regulation […] and if they are, let's say, in software forms, then the issue of premium services comes in.”* “*Do we have policies that can harmonize such […] access to devices?”* “*Why does a motorcycle cost much cheaper than an electric wheelchair? Because if you look at the materials used, they are more or less the same.”* “*They are zero rated when it comes to VAT for hearing aids. Except accessories.”* “*Accessories, obviously, when we bring them in from our headquarters in the U.S., we have to pay VAT.”* “*When we look at option 2, where we are talking about national tax structures […] reducing or doing away with tax for the sake of assistive technology […] is going to really need a lot of mobilization efforts from implementers like us to ensure the policymakers buy into this.”* “*Some of the people decide just to pay the tax because the process is too long. The process of getting the tax exemption is too long.”* *“We need to find out what is it that the UN agencies are doing that their goods are able to be exempted in that short period […] and these other partners, who are also bringing in similar products.”* “*If you want them to have tax exemption, then they needed to understand why they need to exempt some of those products.”* “*The issue of tax is very complicated and very challenging when it comes to Kenya, per se.”*
Option 3	Local production and quality challenges	*“These devices are not produced in the country in a major way, apart from a few wheelchairs.”* “*Those that are produced could not be meeting global standards because they are lacking in terms of the capacity to align with the WHO requirements.”* “*If the local manufacturers have to do the manufacturing, they do not get any incentive. So if they are to produce, the cost would still be very high.”* “*If we could encourage local manufacturing, I think probably by giving incentives to the local producers, that would go a long way in making the assistive devices more available and more affordable.”* *“The issue of local production […] the challenge is […] the quality of the product that is being produced.”* *“We have global standards. When we produce this […] make sure that they're actually quality […] We have standards that have been set by WHO.”* “*You really need to promote local production of assistive products through policy incentives.”* *“If they're produced locally, then […] they can also work out the process of reducing the taxes for us to access them in an easy way.”* *“The current government here is stressing more […] manufacturing as one of its pillars. And I think it's gonna be easy to align this with the current government objective.”* *“Countries […] can take the initiative of coming up with […] projects, or maybe industries that produce some of these things […] other than waiting for people or donors to come to their aid.”*
Option 4	Global procurement can expand access	*“Promoting of partnership and international cooperation […] to expand access. And at the global level, organizations like UNICEF is trying to create a global catalog […] to reduce the cost of assistive products.”* “*Governments […] UN agencies and NGOs and others are actually able to leverage on […] these procurement mechanisms […] to increase access.”* “*There is a global procurement hub […] where UNICEF and WHO are trying to work with the global suppliers […] to renegotiate these prices in terms of market shaping.”* “*In UNICEF catalog, it's actually sold at $115. That price you can never find anywhere globally, and that is part of UNICEF and WHO effort to reduce those prices of those hearing aids.”* “*If the government of Kenya or the government of Egypt want to buy that product through UNICEF, they will get it at that price.”* “*With UNICEF and WHO […] we are also trying to work on supporting suppliers, but also government in terms of promoting the provision of quality assistive products.”* “*Using the UNICEF and WHO and ATscale partnership in terms of also advocating for better policies […] reaching out to governments.”* “*The traditional approach has always been the one that provides assistive technologies or assistive devices through international partnerships and corporations. But this is not sustainable.”*
Option 5		*“Information, awareness regarding assistive devices […] is not readily available to the communities of those who need them.”* “*Affording these devices is not well spelled out, how you can afford them, where you can get them.”* “*As we continue to raise awareness to the society, […] the stigma will also be away from it.”* “*Public awareness is necessary all year, every day, if possible […] awareness cuts across from the community all the way to those who are making decisions.”* “*We have new legislators […] who come into place who do not understand issues of persons with disabilities.”* “*The campaigns with media […] I find option 5 is so workable.”* “*I'm even looking at and seeing how we can plug in as a department and pick some of these activities and quickly put them in our strategic plans and also in our work plans.”* “*The politicians will not engage professionals […] they just go out and buy assistive devices […] and they start giving out wheelchairs, causing more damage to persons with disabilities, because they don't follow the WHO standards of prescribing.”* “*They need to be a lot of public awareness to politicians to avoid this, and to the community not to accept these freebies.”* “*Awareness, creation, and sensitization to everybody, the lawmakers, the administrators, the community, our public servants, the health workers, is also very important.”* “*Public awareness is true […] where we have this blanket distribution of assistive products. No assessment is done, quality issue, no one cares about it. […] They actually cause more harm than good to those beneficiaries.”* “*You provide such a wheelchair to a child with cerebral palsy that will require a more sophisticated wheelchair. And so that actually causes more harm than good. But I think the community have no knowledge about this.”*
Implementation considerations	Governance	*“National Council do try to subsidize some of these devices, like motorized wheelchair. National Council will pay a certain amount.”* *“The policies regarding assistive devices in the country are scrappy. They are not well developed….[]…They are not well disseminated.”* “*We have very poor coordination, weak links of coordination of these essential devices.”* *“You'll find that you are not even going to find, in the Ministry of Health organogram, rehabilitation. It is not anywhere on the organogram. So, you really have to first fight for your space, and now start selling your idea to the people.”* “*There's a lot that we need to be done in terms of ensuring that these adequate policies and guidelines that support and promote the use of assistive devices.”* “*Already we have so many good laws that are in place, and yet we are not implementing […] my thought is not to create another law yet until the ones that we have are actually actualized.”* “*They're involving different sectors, and every different sector has their own idea of how they want to do things, so how to bring them on board and convince them […] is where the big challenge will be.”* “*There are those who make decisions beyond us. And those are the people that we really need to target and convince about these options.”* “*You could have some policies, show them the policies, disseminate their policies. But when it comes to issues of implementation, it is a very hard task, because the counties say that governments by themselves. You cannot force them, or you cannot enforce them where they are not implementing those policies, no matter how necessary or how important they are.”* “*This awareness needs to be done, and it should come all the way from the DPO […] all the way to the communities and the ministry […] so they understand their rights when they are provided with these assistive products.”* “*A lot […] needs to be done in terms of dialogue to ensure that these manufacturers and traders of assistive devices through the entire chain are able to […] be sensitive.”*
	Imported devices are costly to maintain	*“If we get the imported products, some of them, when it comes to issues of repair, maintenance, and service, it's very expensive.”* “*When it comes to the rehabilitation equipment, you find that they are not readily available in the health facilities.”* “*If it does, it will buy the imported one that cannot […] is not usable for all the terrain.”*
	Limited investment restricts supply	*“Manufacturers and investors […] are shy of investing in assistive devices […] because this is not a mass market. And therefore, at some point, it may not be a breaking-even business.”* “*They have formed themselves in form of cartels and will not want to open up some of their technologies so that it can be adaptable and replicated en masse.”* *“The trade institutions do not look at assistive devices as humanitarian action. They see those ones as profits.”*
	Free hearing aids through partnerships	*“We connect with doctors […] in terms of doing medical camps and giving free hearing aids.”*
	Limited understanding hinders implementation	*“Most of the people don't understand the issues disability. They don't understand the issues assistive products, assistive devices, assistive technology.”*
	Leadership lacks technical knowledge	*“Some of the people who are appointed or nominated to head these sections are not medics, they don't have any background, they don't understand anything about medicine, they don't understand anything about disability, they don't understand anything about assistive products and assistive technology.”*
	Hearing aid coverage is restricted	*“It is one device per household per year […] then they can only get one hearing aid that is covered by the Social Health Authority.”*
Equity considerations	Independent living	*“So that at least we are assisted, so that we continue living independently like other people in the world.”*
	Digital costs widen inequalities	*“If one can't access the premium service, and the other person access the premium service, are we not widening the financial gap between these two individuals?”*
	Marginalized groups face greater exclusion	*“Those who can afford them are able to use them. And those who can't, then definitely they continue to be marginalized.”*
	Geographic and financial exclusion	*“We, as digital disability advocates, sometimes we move to marginalized areas, like urban slums, or rural areas […] because of the cost of Braille […] they are not able even to access digital platforms.”* “*The available may be mostly in the city, and if you look at the local or the rural areas, the assistive devices are not available.”* “*Persons with disabilities [are] being referred to areas that are even very far away from where they reside, hence some of them do not have the cost to cater for that.”*
	AT access is a right	*“It's not that I'm doing you a favor, I think it should come from […] a rights-based approach.”* “*We have these policies in place that ensures that […] equal access when it comes to services for persons with disabilities.”* *Multiple disabilities compound disadvantage: “I don't have any assistive device or devices, rather, which can help me do my work properly and remain equal to other people who do not have these disabilities.”* “*I'm wondering how I get these assistive devices that would assist me live and do things normally, like any other person without other people assisting me.”* “*Women with disability have more challenges and acceptance in the community than the male counterparts with disability.”*
	Device appearance influences acceptance	*“There are those that are too big, and some of the youths who are using them […] don't feel comfortable having them.”* “*The cosmetic appearance of any assistive device is very key for the user.”* *“If it is a child, you would find that more bright, colorful prosthesis appeals to a child […] If it is a teenager, they are very sensitive about how they look.”* “*She stopped using her hand splint because it wasn't looking appealing.”*
	Culture and beliefs affect access	*“We have some ethnic groups that still believe that disability is a curse […] or a punishment from a higher power.”*
	Language barriers limit communication	*“They're not able to express their needs well.”* *“We don't have enough […] sign language communication.”*
Dialogue guidelines	*“I feel like all these options can work […] on paper, it's easy to say that the options do work. But when it comes to the reality of it, very different organizations […] are contributing something to ensuring that assistive technologies can be accessed by all persons with disabilities.”* “*Even assistive devices should be treated as essential products.”*	

Source: Author's own elaboration.

## Discussion

The DD highlighted key issues related to access to AT, including difficulties in access, the high cost of devices, the weakness of tax exemptions, territorial inequalities, and coordination challenges among the different actors involved. Although national contexts have particularities, the discussions revealed common barriers that compromise policy implementation and the promotion of equity in access.

In Mozambique, the deliberative dialogue brought together different actors involved with AT and confirmed the relevance of the options presented, especially the reduction of tariffs and trade barriers and the strengthening of local and regional production. Among the main challenges discussed were the high cost of devices, the low effectiveness of tax exemptions, and the lack of monitoring mechanisms to ensure that the benefits actually reach users (16, 17). Participants also observed that issues such as malaria and tuberculosis often dominate the health agenda, leaving AT in the background. Despite limitations in local production, opportunities were identified in maintenance and refurbishment workshops, reinforcing the importance of industrial policies linked to public procurement and co-creation processes with users (18).

There was consensus on the need for training in the prescription, adaptation, and monitoring of devices, with emphasis on the integration between primary care and rehabilitation services, in accordance with the Community-Based Rehabilitation guidelines (19). Rural and remote areas were identified by participants as priority settings for improving access and decentralizing AT services. Final recommendations included campaigns against stigma, active outreach in partnership with Organizations of People with Disabilities (ODDs), and the improvement of information systems, with data disaggregated by gender, income, and territory (20).

In Chad, the dialogue, although it only included representatives from that country—despite an invitation also extended to Congo—promoted a rich exchange between institutional, technical, and community actors. Discussions highlighted the importance of awareness campaigns and advocacy, international cooperation, and intersectoral partnerships as initial steps to enable fiscal reforms and boost local production. Participants emphasized the need to create a national database and a tax code for TA products, seen as an essential condition for the effectiveness of policies (2). The risk of “private capture” of tax exemptions was also discussed, where tax benefits are not reflected in prices for users (21). The Central Purchasing Office was cited as a strategic arrangement to integrate procurement, distribution, and technical information processes, as guided by the WHO Model Quality Assurance System for Procurement Agencies (22). The debate broadened the concept of equity, incorporating dimensions such as stigma, lack of information, and institutional fragmentation, and highlighted the shortage of specialized professionals as a significant bottleneck—a challenge also recognized in the Global Report on AT (2).

In the Deliberative Dialogue between Kenya and Egypt, distinct but complementary challenges emerged.In Egypt, discussions revealed high costs, gaps in insurance coverage, reliance on charitable support, and regional inequalities in access to AT, particularly between major urban centers and rural areas. Participants emphasized the importance of cooperation between government and healthcare providers, international partnerships, professional training, and mechanisms to guide persons with disabilities in accessing available rights and services.. In Kenya, the deliberations revealed multiple and interconnected barriers, including limited insurance coverage, high out-of-pocket costs, dependence on imported devices, weaknesses in the implementation of tax exemptions, limited local production, and coordination challenges. Cultural beliefs, stigma, communication barriers, gender inequalities, multiple disabilities, and the physical appearance and acceptability of assistive devices also emerged as important equity considerations. Participants emphasized public awareness, inclusive and user-centered design, quality standards for local production, stronger implementation of existing policies, and international cooperation as important strategies for expanding equitable and sustainable access toAT.

### Knowledge translation as an integrating axis

Multilingual deliberative dialogues highlighted the translation of knowledge as a central axis of deliberations, allowing for the contextualization of global evidence within national scenarios, reducing duplication, optimizing resources, and promoting comparability between countries. The dialogues functioned as spaces for contextualized co-production, combining scientific evidence, professional experiences, community knowledge, and user experiences, in accordance with the principles of deliberative dialogues (11, 12).

### Key barriers to accessing assistive technologies

Despite contextual differences, convergent patterns of barriers to access to AT emerged. Economic barriers were transversal, with the high cost of devices persisting even where tax exemptions exist, due to the low effectiveness of policies, communication failures, lack of monitoring, and the risk of “private capture” of tax benefits (21). These findings reinforce the need for regulatory and transparency mechanisms so that tax reforms translate into greater equity.

The fragility of local production was also recurrent, marked by limitations in the production, adaptation, and maintenance of AT), difficulties in ensuring quality standards, and unstable partnerships. While some countries highlighted the potential of regional workshops and refurbishment, others emphasized the cultural and aesthetic suitability of the devices, corroborating evidence that supports industrial policies linked to public procurement and co-creation with users (18).

The shortage of specialized human resources has compromised both the safe and continuous use of AT at the individual level and the systemic capacity for policy implementation, as also highlighted in the Global Report on AT (2). Furthermore, weaknesses in information systems and governance, including gaps in databases and national assistive product lists, as well as ineffective coordination among relevant institutions and sectors, have limited policy implementation (16). Gaps in data on access to AT also hinder the identification and monitoring of inequalities (23).

### Contextual specificities and implications for policies

The dialogues also revealed relevant national specificities. Mozambique highlighted geographic barriers, fragile local production, and the need for greater integration between primary care and rehabilitation services; Chad emphasized governance, advocacy, limited specialized workforce, and the initial structuring of the AT system; Egypt highlighted territorial inequalities, financial barriers, gaps in insurance coverage, and the importance of trained health workers; and Kenya emphasized economic, cultural, communicational, and symbolic barriers, including stigma, gender inequalities, and the social acceptability and suitability of assistive devices.

Participants highlighted the role of international cooperation.in reducing costs, ensuring quality, and promoting access to AT as a human right, through global catalogs, technology transfer, and strategic procurement.

### Summary for the global AT agenda

Taken together, the dialogues indicate common challenges—dependence on imports, informational fragility, scarcity of specialized training, territorial inequalities, and weak intersectoral governance—but also clear opportunities for progress, such as monitored tariff reforms, strengthening local production with quality standards, integrating ATs into primary care, rehabilitation, and universal health coverage, anti-stigma campaigns co-produced with organizations of persons with disabilities, and strategic international partnerships.

## Final considerations

The deliberative dialogues held in Mozambique, Chad, Egypt, and Kenya show that, despite cultural, institutional, and economic differences between countries, common structural barriers persist that limit equitable access to AT. Among these barriers are the high cost of devices, weaknesses in the implementation and monitoring of tax exemptions, fragile local production, shortages of specialized professionals, insufficient standardized data, and gaps in institutional and intersectoral coordination. These findings reinforce that AT policies cannot be fragmented or *ad hoc*, but should integrate strategies involving economic regulation, capacity building, quality-assured local production, community engagement, and robust information systems.

The use of a synthesis of evidence adapted to different national contexts demonstrated the power of knowledge translation as a tool to align global evidence with local realities. The dialogues enabled managers, professionals, organizations of persons with disabilities, and specialists to jointly discuss relevant and feasible policy options, favoring the identification of context-specific priorities, implementation considerations, and areas of convergence between technical, institutional, and community perspectives.

Overall, the findings indicate that advancing the AT agenda in the African countries analyzed depends on a combination of four central axes: (1) simplifying bureaucratic and customs procedures and strengthening the implementation of tax exemptions and reductions so that fiscal benefits effectively reach end users; (2) strengthening local production, adaptation, and maintenance in accordance with appropriate quality standards; (3) integrating AT into primary care, rehabilitation, and universal health coverage strategies; and (4) ensuring the active participation of persons with disabilities and their organizations, thereby promoting social legitimacy, cultural appropriateness, and responsiveness to users’ needs.

Thus, the results of the Deliberative Dialogues not only reaffirm the relevance of the synthesized evidence but also provide a foundation for planning subsequent implementation steps and informing policy and technical decisions that consider the specificities of each context. Incorporating these options into national agendas could contribute to more equitable, sustainable, and people-centered AT systems, translating knowledge into concrete action and strengthening international commitments to the inclusion and rights of persons with disabilities.
